# The potential of targeted IL-4 therapy for the comorbidity of atopic dermatitis and attention-deficit/hyperactivity disorder in children via the skin-brain axis

**DOI:** 10.3389/falgy.2026.1887240

**Published:** 2026-07-10

**Authors:** Yingjie Zhou, Yuan Sun, Weisheng Hu, Qing Wang, Zijia Chen, Ning Huang, Lingling Li

**Affiliations:** 1 Department of Dermatology, The First Clinical Medical College of Beijing University of Chinese Medicine (Dongzhimen Hospital), Beijing, China; 2 Fujian Provincial Key Laboratory of Integrative Dermatology, The Second Affiliated Hospital of Fujian University of Chinese Medicine, Fuzhou, Fujian, China; 3 Department of Pediatric, Dongfang Hospital, Beijing University of Chinese Medicine, Beijing, China; 4 College of Chinese Medicine, Chengdu University of Traditional Chinese Medicine, Chengdu, Sichuan, China; 5 Department of Dermatology, Chongqing Hospital of Traditional Chinese Medicine, Chongqing, China; 6 Department of Dermatology, The Second Affiliated Hospital of Fujian University of Chinese Medicine, Fuzhou, Fujian, China; 7 Department of Dermatology, Dongzhimen Hospital, Beijing University of Chinese Medicine, Beijing, China

**Keywords:** atopic dermatitis (AD), attention-deficit/hyperactivity disorder (ADHD), children, IL-4, skin-brain axis (SBA)

## Abstract

Although Atopic Dermatitis (AD) and Attention-Deficit/Hyperactivity Disorder (ADHD) frequently coexist in children, the underlying mechanisms remain unclear. The “Skin-Brain Axis”(SBA) hypothesis, based on the shared ectodermal origin of the skin and the central nervous system(CNS), provides a theoretical foundation for communication between AD and ADHD. However, standardized biomarkers and direct clinical validation are currently lacking. Interleukin-4 (IL-4), a key cytokine driving skin inflammation in AD, may theoretically influence CNS development via the SBA, thereby contributing to the pathophysiology of ADHD; but this hypothesis has not yet been validated in humans. Based on the SBA, the review integrates the dual role of IL-4 in the pathogenesis of AD and ADHD, explores the possibility of IL-4 acting as a bridging signal between peripheral skin inflammation and neurodevelopmental abnormalities, and summarizes existing clinical and preclinical evidence for IL-4-targeted therapies for AD-ADHD comorbidity, while also highlighting current gaps in evidence and limitations in application. Future research should include prospective clinical trials with neurocognitive assessment and brain imaging as endpoints, using direct neuroimmune effects and indirect effects (such as itch relief and improved sleep) as reference indicators, to systematically evaluate the feasibility of IL-4 inhibitors in controlling neurobehavioral symptoms and provide a practical research pathway for clinical translation.

## Highlights

1. Novel Integration of Theoretical Frameworks: This study innovatively applies the concept of the “Skin-Brain Axis” (SBA) to contextualize the comorbidity of atopic dermatitis (AD) and attention-deficit/hyperactivity disorder (ADHD).
2. In-depth Mechanistic Elucidation: By synthesizing current literature, this review explores how IL-4 potentially mediates bidirectional crosstalk between the peripheral and central nervous systems (CNS), providing a molecular bridge for interventions targeting this comorbidity.
3. Translational Clinical Value: This work provides a theoretical rationale for the cross-indication application of biologics and advances the development of precision treatment strategies for affected pediatric populations.

## 1 Introduction

Atopic dermatitis (AD) is a common chronic inflammatory skin disease affecting 15%–20% of children worldwide (1), with approximately half of moderate-to-severe cases persisting into adulthood (2). Characterized by intense itching and impaired skin barrier function, this condition not only severely compromises the children's life quality, but may also be associated with an increased risk of neurodevelopmental comorbidities such as attention-deficit/hyperactivity disorder (ADHD) and Autism Spectrum Disorder (ASD) (3), resulting in a significant health and economic burden (4). ADHD is one of the most common neurodevelopmental disorders in childhood, with a global prevalence of approximately 7.2% (5), primarily characterized by inattention, hyperactivity, and impulsivity. Epidemiological studies show a close comorbidity between AD and ADHD, with the prevalence of ADHD among children with AD reaching up to 11.25% (6), and the onset of AD often preceds that of ADHD, suggesting that early-stage skin inflammation may influence subsequent neurodevelopment (7).

The biological basis for this comorbidity can be explained by the “Skin-Brain Axis” (SBA) theoretical framework, which is a still-evolving conceptual model describing the interactions between the skin and the central nervous system (CNS) via immune, endocrine, and neural pathways (8). If this axis exhibits functional plasticity during critical periods of childhood development, peripheral inflammatory signals may be more readily transmitted to the CNS through immature barrier systems and may exert lasting effects on the formation of neural circuits via epigenetic modifications, synaptic transmission, and neurotransmitter metabolism (9). Therefore, identifying and blocking key signaling molecules that link the periphery to the CNS within the SBA holds significant theoretical importance for understanding the pathogenesis of mechanisms underlying AD-ADHD comorbidity and for developing early intervention strategies.

Recent studies suggested that interleukin-4 (IL-4) is one of the potential candidate mediators linking peripheral inflammation to CNS developmental abnormalities. As a key cytokine in the Th2-type immune responses, IL-4 is not only a central mediator driving skin barrier dysfunction and inflammation in AD, but its elevated levels have also been identified in epidemiological studies as an independent risk factor for ADHD development in AD children (6). This finding suggests that excessive IL-4 exposure may establish a link between peripheral allergic inflammation to central neurodevelopmental abnormalities via the SBA. Physiological levels of IL-4 exert protective effects on cognitive function (10). However, chronic systemic IL-4 elevation associated with AD could theoretically cross the Blood-Brain Barrier (BBB) or be locally induced within the brain, thereby disrupting neurotransmitter homeostasis and affecting synaptic pruning, thereby potentially contributing to the abnormal development of ADHD-related neural circuitry (11, 12). However, this causal chain is primarily based on animal models and indirect evidence, and its direct validation in children with AD remains to be conducted.

Based on the above theory, we propose the following scientific hypothesis: during the period of developmental plasticity period of the SBA in children, AD-associated IL-4 elevation may not only exacerbate skin lesions by compromising the skin barrier but also act as a key signaling molecule that influences the CNS directly or indirectly, thereby potentially inducing or exacerbating ADHD-related symptoms. Therefore, targeting IL-4 or its downstream signaling pathways may represent a novel approach for early intervention and improvement of the comorbidity of AD and ADHD.u.

## 2 Literature retrieval strategies and evidence evaluation criteria

### 2.1 Search strategy

A systematic search was conducted in PubMed, Web of Science, Embase, and the Cochrane Library, with the search period extending to February 2026. A combination of MeSH terms and free-text keywords was used, with core search terms covering three concept groups: (1) atopic dermatitis (“Atopic Dermatitis” OR “Eczema”); (2) neurodevelopmental disorders (“ADHD” OR “Neurodevelopmental Disorders”); (3) Mechanism targets (“IL-4” OR “SBA” OR “Neuroinflammation”). The search was supplemented by hand-searching reference lists.

### 2.2 Inclusion criteria

(1) Epidemiological studies on the comorbidity of AD and ADHD; (2) Studies on mechanisms related to IL-4 and the SBA (including *in vitro* and *in vivo* experiments); (3) Clinical or real-world studies on IL-4-targeted therapies for AD and neuropsychiatric comorbidities; (4) Original research, systematic reviews, or high-quality narrative reviews published in English or Chinese.

### 2.3 Exclusion criteria

Studies for which full-text access was unavailable, non-peer-reviewed preprints, and case reports with weak thematic relevance.

### 2.4 Evidence grading

Animal studies are labeled only as “preclinical evidence”; human mechanistic data are defined as “observational association evidence”. Prospective cohort and interventional studies are used for causal inference. Conflicting data are reported without bias, and sources of heterogeneity are analyzed from three dimensions: anatomical spatial heterogeneity, sample characteristics and disease stage, and methodological limitations.

## 3 Skin-Brain axis

The “Skin-Brain Axis” (SBA) is a theoretical framework describing the bidirectional regulatory relationship between the skin and CNS (13). Both the skin and CNS originate from the ectoderm (14) and exchange information via nerve fibers, immune cells, endocrine signals, and inflammatory mediators (8). Unlike the well-studied gut-brain axis, the SBA currently lacks a standardized evaluation system and a physiological gold standard validated directly in humans. During childhood, the SBA exhibits the unique characteristics of immaturity and plasticity. The period from 0 to 6 years is considered a critical window for plasticity: during this stage, skin nerve endings are densely distributed, the BBB is not yet fully mature, the hypothalamic-pituitary-adrenal (HPA) axis is highly reactive, and microglia are in an active, plastic state, all of which theoretically enhance the efficiency of CNS transmission of peripheral signals. Animal studies indicated that abnormal inflammatory signals can regulate neurodevelopment-related genes (such as Dopamine Receptor D4, Catechol-O-Methyltransferase) through epigenetic modifications, thereby influencing the formation of neural circuits. However, these findings primarily stem from preclinical models, and there is currently a lack of direct evidence to confirm whether they hold true in children with ADHD or whether they lead to irreversible tissue damage related to ADHD. Nevertheless, the relatively short repair window between ages 3 and 6 suggests that intervention during this stage might partially reverse these abnormal changes, providing a theoretical basis for early intervention (15).

In the pathological context of AD complicated by ADHD, SBA may theoretically serve as one of the pathway linking peripheral skin inflammation to the CNS unctional changes. Cytokines (particularly IL-4) generated by skin barrier disruption and local inflammation in AD may enter the brain via the circulatory system or trigger a CNS immune responses by activating afferent nerve endings (16). If such peripherally initiated CNS neuroinflammation occurs during the critical window for neural circuit development, it could theoretically affect the development of dopaminergic circuits in ADHD-related brain regions such as the prefrontal cortex and striatum (17).

**Figure 1:**
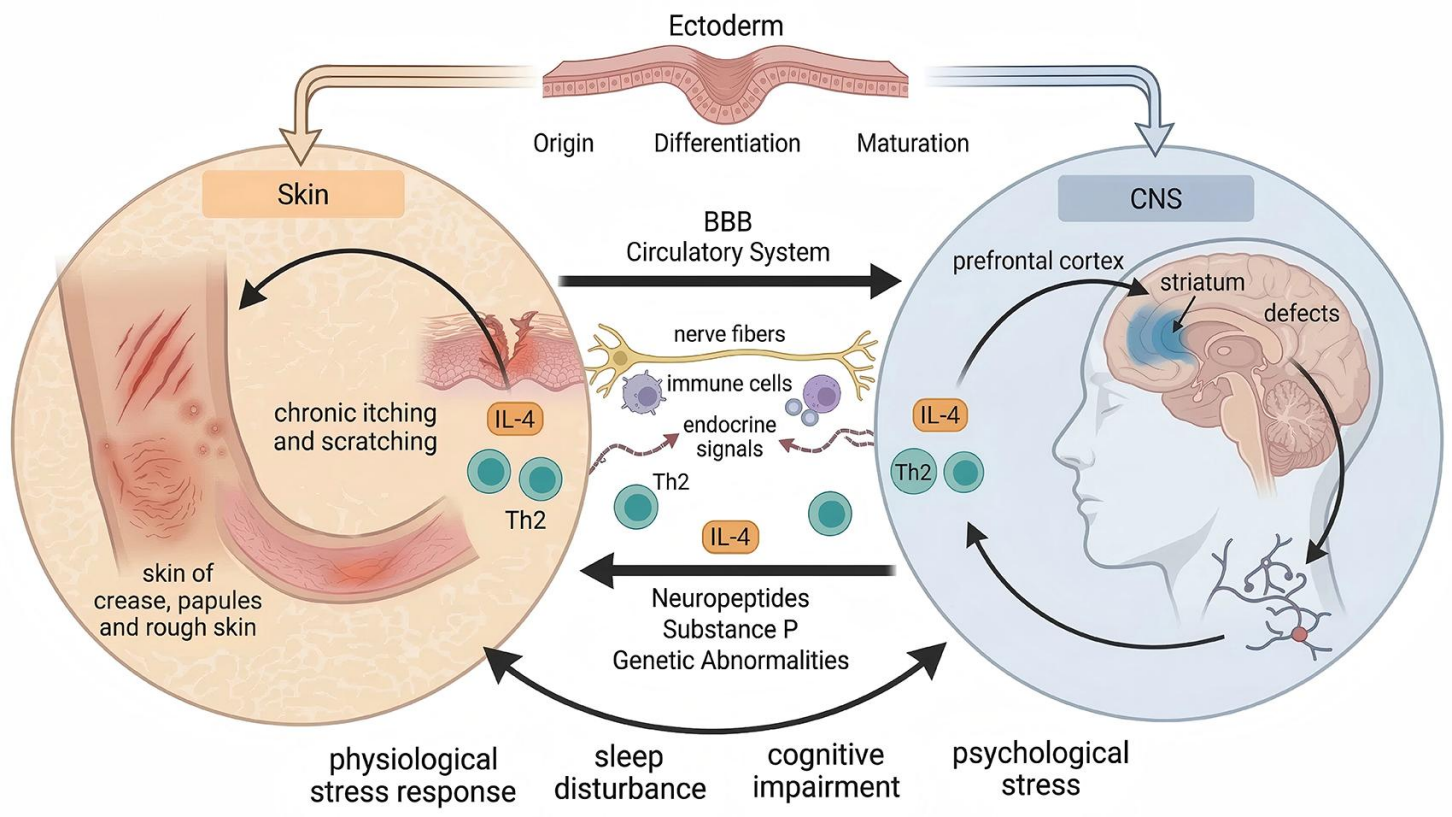
Schematic illustration of the SBA crosstalk mechanism.

Conversely, ADHD-related behavioral abnormalities (such as intense scratching) and psychological stress (such as anxiety from social difficulties) may feed back to activate skin nerve endings, triggering the release of neuropeptides such as substance P, which further amplifies local Th2-type inflammation. Theoretically, this may constitute a positive feedback mechanism (18). Current research on SBA still faces core limitations: firstly, the lack of non-invasive biomarkers to quantitatively assess its function; secondly, there is a lack of direct *in vivo* evidence regarding the transmission of cutaneous inflammatory signals to CNS; Thirdly, the dynamic characteristics of SBA across different developmental stages in children have not yet been systematically elucidated. Most of the evidence for this bidirectional “skin-brain” interaction still comes from animal experiments and indirect observations; direct validation in children with comorbid conditions and the establishment of causal relationships require further research (Figure 1).

Figure 1: Skin and CNS share a common ectodermal origin and form a bidirectional regulatory network via the circulatory system, nerve fibers, and endocrine signals. Skin inflammation, characterized by Th2 immune responses and IL-4 secretion, can disrupt central functions by crossing the BBB, leading to cognitive impairment and sleep disturbance. Conversely, central stress and neuropeptides (e.g., Substance P) exacerbate skin itching and inflammation in return, establishing a pathological skin–brain vicious cycle that underlies the comorbidity between skin disorders and neuropsychiatric symptoms.

## 4 The role of IL-4 in the pathogenesis of AD and ADHD

### 4.1 Biological sources, transmission, and effects of IL-4

IL-4 is a core cytokine of Th2-type immune responses, primarily produced by activated Th2 T lymphocytes, although it can also be secreted by mast cells, basophils, and other cells. The biological effects of IL-4 are initiated through binding to its specific receptors, which comprise the type I receptor (composed of IL-4R*α* and the common gamma chain *γ*c) and the type II receptor (composed of IL-4R*α* and the IL-13R*α*1 chain) (19). Upon activation, IL-4 receptors primarily transmit signals through the Janus kinase (JAK)-signal transducing and activator of transcription (STAT) pathway. Phosphorylated STAT6 forms dimers that translocate into the nucleus to regulate the transcription of target gene (20, 21), exerting immunomodulatory functions such as driving Th2 cell differentiation, promoting the production of IgE antibodies by B cells, and inducing the polarization of macrophages toward the M2 phenotype (22). Furthermore,IL-4 in the skin not only directly suppresses the expression of key barrier structural proteins such as filaggrin (FLG) and loricrin (LOR) in keratinocytes (KC) (23, 24) but also inhibits fatty acid elongase (ELOVL) expression via the JAK/STAT signaling pathway (25, 26) and regulates sex hormone synthesis mediated by 3*β*-hydroxysteroid dehydrogenase 1 (HSD3B1) (27). These mechanisms ultimately alter lipid metabolism, thereby impairing skin barrier function. Under pathological conditions, IL-4 can influence the CNS through two pathways: crossing the BBB and modulating microglia. This conclusion is primarily supported by evidence from animal studies: when BBB permeability increases, elevated peripheral IL-4 can directly permeate into the brain parenchyma (28), or be locally produced by immune cells in the meninges and choroid plexus (29). On the other hand, IL-4 promotes the polarization of microglia toward the neuroprotective M2 phenotype by regulating their morphology and function, thereby participating in neurogenesis and synaptic remodeling Normal levels of IL-4 are crucial for hippocampus-dependent learning, memory, and synaptic plasticity (30). Notably, IL-4 expression and regulation in pediatric tissues exhibit significant developmental specificity, which is considered the molecular basis for children's potential increased sensitivity to IL-4-mediated inflammatory effects (31).

### 4.2 The role of IL-4 in the pathogenesis of pediatric AD

IL-4 levels are significantly elevated in patients with AD (32), and its effects are primarily manifested in driving and maintaining Th2 immune polarization, directly impairing skin barrier function, and contributing to the onset and persistence of itching. Previous studies have demonstrated that IL-4 drives the differentiation of naive T cells into Th2 cells and forms a positive feedback loop through autocrine IL-4 and high expression of IL-4R*α*. It synergizes with IL-13 to activate the JAK/STAT signaling pathway, thereby amplifying and sustaining the Th2 response (33). Other studies have also found that IL-4 can directly downregulate the expression of key barrier proteins such as filaggrin (FLG), disrupting KC integrity and hydration (24), which leads to physical barrier damage, resulting in dry skin and itching (34, 35); simultaneously, it reduces the production of antimicrobial peptides from KCs, disrupts the skin's chemical barrier, and induces dysbiosis of the skin microbiome, leading to bacterial skin infections such as Staphylococcus aureus (36). This barrier disruption makes the skin more susceptible to invasion by environmental allergens and microorganisms, which in turn continuously activates the immune system, creating a vicious cycle of “inflammation and barrier dysfunction” that complicates the condition and leads to chronicity. Overexpressed IL-4 directly activates sensory neurons via the shared IL-4R*α* receptor, promoting sensitization pathways such as increased expression of transient receptor potential ankerin 1 (TRPA1), enhanced itch signaling, epidermal nerve growth, barrier dysfunction, and inflammatory cell recruitment (37), increasing neurons responsiveness to itch-inducing substances in inflamed or barrier-compromised skin and generating amplified itch response. Concurrently, IL-4 can induce KCs to produce alarmins such as thymic stromal lymphopoietin (TSLP), IL-25, and IL-33 (38). These alarmins directly activate sensory neurons, triggering intense activation of local neurogenic mast cells in the skin, leading to the release of mediators such as histamine and leukotrienes (18), which further recruits and activates Th2 cells, inducing scratching behavior; on the other hand, they activate ILC2s and dendritic cells to secrete more pruritus-inducing factors, including IL-4, driving the persistent itch-scratch cycle (39). This state of low-grade inflammation, caused by persistent skin inflammation and impaired barrier function, also significantly increases the risk of AD patients developing other atopic conditions such as asthma and allergic rhinitis (40). The developmentally low expression of FLG in pediatric epidermal KC not only directly exacerbates the severity and chronicity of AD but also creates a permissive environment for persistent allergen penetration and systemic immune dysregulation, thereby significantly elevates the risk of subsequent atopic diseases such as asthma and allergic rhinitis, as well as neuropsychiatric comorbidities.

### 4.3 Pathological link between IL-4-mediated neuroinflammation and pediatric ADHD

It is currently believed that ADHD results from a neuroimmune dysfunction caused by the interaction of multiple factors, including genetic, environmental, and immune factors (41), and that IL-4-mediated immune responses may affect neurological development through chronic low-grade neuroinflammation, leading to the pathophysiological process of ADHD. This section presents relevant research findings stratified by the level of evidence.

#### 4.3.1 Observational evidence in humans

Clinical observations show that serum IL-4 are significantly elevated in patients with ADHD (42), and are correlated with the severity of behavioral symptoms (43). However, early studies found that IL-4 levels in the cerebrospinal fluid (CSF) of children with ADHD were conversely lower than those of TNF-*β* (44). This discrepancy between peripheral and central levels may stem from multiple factors: anatomically, in the absence of severe BBB leakage high peripheral concentrations of IL-4 may be retained in meningeal vessels or the choroid plexus, or may indirectly modulate the central microenvironment via peripheral immune cells (29), rather than circulating freely large quantities in the deep CSF. Methodologically, detection sensitivity, sample processing, and confounding factors from comorbidities may also influence the quantitative results of CSF cytokines. Currently, there remains a lack of dynamic imaging and pathological confirmation regarding how peripheral IL-4 penetrates the brain at specific pathological stages and its dynamic distribution patterns across different anatomical compartments (28). The aforementioned findings only demonstrate a statistical association between serum IL-4 and ADHD and cannot rule out the interference of confounding factors; therefore, a causal relationship cannot be established.

#### 4.3.2 Evidence from animal models

Studies using rodent models of ADHD have shown significantly elevated levels of IL-4 in key brain regions such as the hippocampus. In these models, pathological levels of IL-4 can penetrate the compromised BBB, interfere with the microglia-mediated process of neuronal synaptic pruning, and impair their clearance capacity, providing a structural-level theoretical basis for behavioral phenotypes such as attention deficits (11). Furthermore, in animal models of neurological inflammation or injury, IL-4 has been observed to regulate the dopamine and norepinephrine systems, leading to an imbalance in neurotransmitter levels (45–47). Physiological levels of IL-4 can modulate the phenotype of meningeal myeloid cells and the expression of neurotrophic factors, thereby maintaining adaptive immunity and normal cognitive function (48). These findings provide a theoretical basis at the mechanistic level for IL-4's involvement in neurodevelopmental regulation. However, they all originate from preclinical models, and their extrapolation to humans remains unclear. Significant species heterogeneity exists between humans and rodents in terms of the developmental timing of brain regions, the maturation patterns of the BBB, and the functional phenotypes of microglia. Therefore, conclusions from animal experiments cannot be directly extrapolated to pediatric patients.

Based on the above findings, we hypothesize that when peripheral IL-4 concentrations in children with AD remain persistently elevated and exceed physiological thresholds, IL-4 may enter the CNS through a compromised BBB or indirectly influence the CNS microenvironment. If central exposure is prolonged, it could theoretically have adverse effects on the development of neural circuits. However, it must be clarified that this complete causal chain is primarily derived from animal models and indirect evidence and has not been directly validated in children with AD and comorbid ADHD. Whether IL-4 crosses the human BBB at biologically effective concentrations and whether it directly causes ADHD-related neural circuit defects remains to be confirmed by prospective studies.

## 5 IL-4 influences AD with comorbid ADHD via the SBA

Based on existing preclinical and observational evidence, we propose that IL-4 may mediate the bidirectional comorbidity of AD and ADHD via the SBA. However, the strength of evidence varies across different pathways, and the overall model requires prospective clinical validation.

### 5.1 The potential role of IL-4 in inducing or exacerbating ADHD in AD

Childhood is a critical period for neural circuits remodeling and the establishment of immune barriers. Clinical epidemiological evidence suggested that persistently elevated IL-4 levels in the peripheral circulation are one of the high-risk factors for ADHD in children with AD (6). Regarding how peripheral skin inflammation exerts cross-systemic effects on CNS behavior, existing research primarily points to two possible pathways: direct neuroimmune transmission and indirect somatic mediation. With regard to direct neuroimmune transmission, studies in rodents have shown that sustained peripheral immune activation can damage BBB and increase its permeability, facilitating the infiltration of peripheral IL-4 into CNS, which can directly interfere with the synaptic pruning process mediated by microglia (49). Given that the prefrontal-striatal circuit in human development also relies on microglia for activity-dependent synaptic pruning, we hypothesize that IL-4 entering CNS may also influence the development of cognitive brain regions in human children through a similar mechanism, thereby contributing to ADHD phenotypes such as hyperactivity and impulsivity (11, 12). However, this hypothesis is primarily based on animal models and requires validation through future human neuroimaging studies and prospective cohort studies.

In terms of indirect somatic mechanisms, severe skin lesions mediated by IL-4 and intractable nocturnal pruritus often lead to long-term disruption of sleep architecture in affected children. Multiple large-scale clinical studies have demonstrated that this chronic sleep deprivation, resulting from peripheral somatic symptoms, although not the primary cause of neurodevelopmental disorders such as ADHD, is an independent risk factor that exacerbates existing neurocognitive impairments and worsens core ADHD symptoms, including inattention and impaired impulse control (50). Therefore, AD-derived abnormal IL-4 may act as a pathological signaling molecule that disrupts CNS microenvironment, while also indirectly worsens cognitive performance by inducing sleep deprivation; this synergistic effect provides an explanation for how AD exacerbates ADHD symptoms. It should be noted that evidence for the core mechanisms of the direct neuroimmune pathway primarily comes from animal experiments, whereas indirect pathways such as sleep deprivation and psychological stress have more robust observational clinical support; the relative contributions of these two pathways to the onset and progression of comorbidity in humans remain unclear.

### 5.2 The potiential role of IL-4 in ADHD-induced or aggravated AD

In addition to the transmission of peripheral inflammation to the CNS, ADHD-associated neural stress and behavioral abnormalities may reciprocally affect cutaneous IL-4 expression in skin tissues via descending pathways, contributing to AD persistence or exacerbation. Children with ADHD are chronically subjected to high levels of neurocognitive and emotional stress, which significantly induces hyperactivity of HPA axis and sympathetic nervous system (51). Persistent neuroendocrine dysregulation triggers the massive release of neuropeptides (e.g., substance P and calcitonin gene-related peptide) via cutaneous nerve endings. These mediators directly activate local mast cells in the skin and induce Th2 cells to overexpress IL-4, thereby amplifying local allergic reactions and neurogenic inflammation (52). Behaviorally, impulsivity may lead to more vigorous scratching responses (39). Compared with only AD children, those with co-occurring ADHD may have a lower itch perception threshold and exhibit more intense and frequent scratching. This persistent and intractable itch-scratch cycle may further exacerbate skin barrier damage, indirectly contributing to the accumulation of local IL-4 and the amplification of inflammation. However, there is currently a lack of prospective studies directly assessing the dynamic changes in IL-4 levels in children with ADHD following exacerbate impulsive behavior; the aforementioned negative regulatory pathway remains to be experimentally verified.

### 5.3 Additional potential pathways for IL-4 in AD with comorbid ADHD

Multioomics evidence indicated that early gut microbiota dysbiosis in children with AD may drive Th2 cell polarization and excessive IL-4 secretion; abnormally accumulated IL-4 may disrupt BBB integrity via the Gut-Brain-Skin Axis (GBSA) and induce central penetration (53), forming a cross-systemic inflammatory cascade (54), offering a perspective on GBSA involvement in ADHD co-occurring with AD. Clinical multi-omics analyses further revealed that the abundance of beneficial bacterial populations producing short-chain fatty acids (SCFAs) in the gut microbiota of children with ADHD is significantly reduced (55). As key gut-derived neuroprotective factors, SCFAs have been shown to participate in maintaining the integrity of tight junctions in the BBB and to regulate the maturation and physiological functions of microglial (56). When SCFA-mediated immunoregulatory protection is compromised, microglia may become hyper-responsive to IL-4 triggering abnormal immune responses and deviant synaptic pruning that may affect prefrontal-striatal and hippocampal circuits development (57). Preclinical studies have shown that GBSA-targeted interventions correcting dysbiosis can reduce peripheral IL-4 and systemic the inflammation, with associated improvements in allergic skin lesion-related behavioral and cognitive deficits observed in animal models (58). At the same time, a deficiency in gut microbiota metabolites may also exacerbate the CNS's exposure to systemic inflammation. However, these mechanisms are largely based on animal experiments and indirect evidence; validation in human comorbid patients remains an open question.

**Figure 2:**
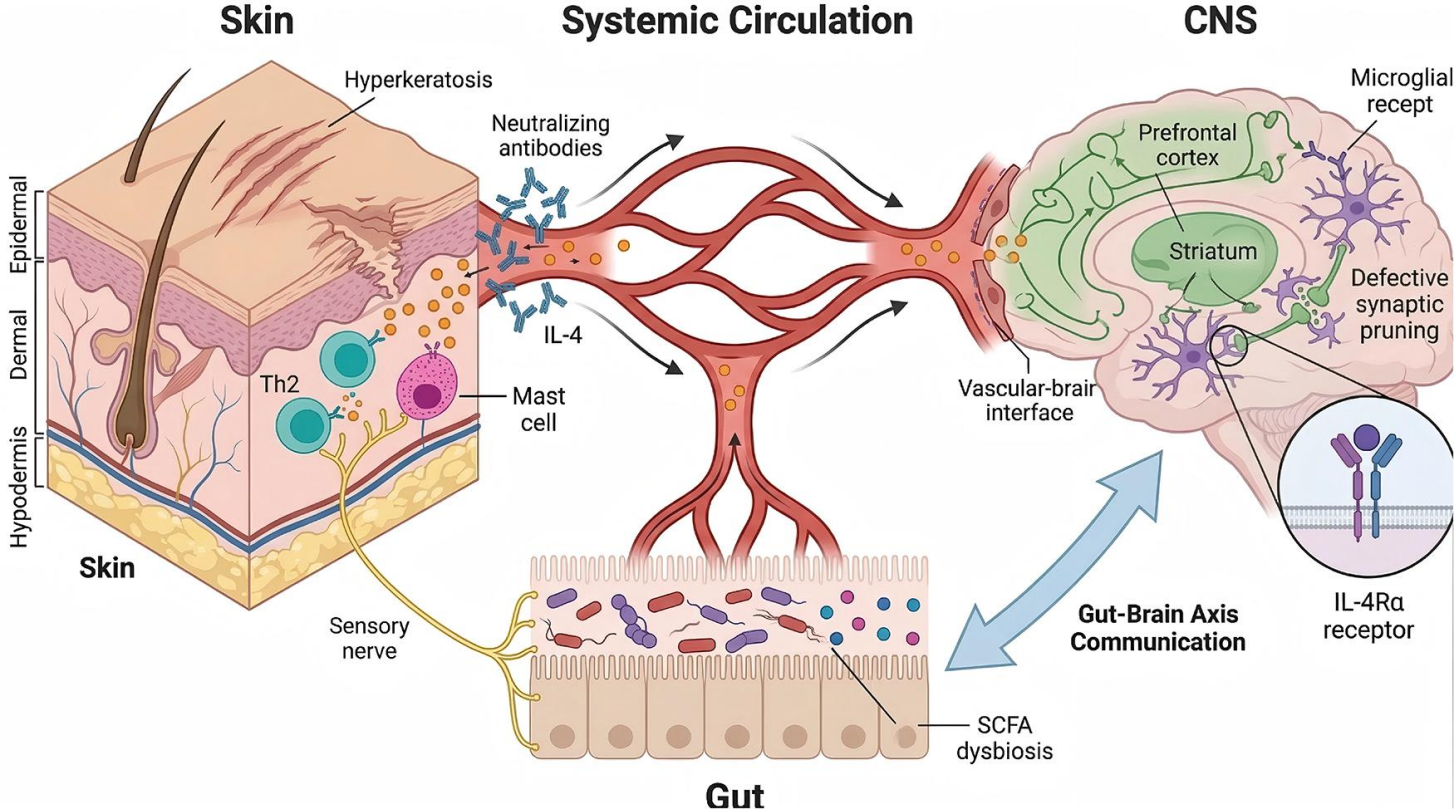
Diagram of the SBA mechanism of comorbidity between AD and ADHD.

In summary, AD-derived IL-4 elevation may contribute to ADHD pathophysiology through multidimensional pathways: (1) Animal studies suggest that IL-4 may penetrate the compromised BBB and interfere with microglia-mediated synaptic pruning, providing a structural basis for abnormalities in CNS circuits development; (2) In models of neurological inflammation, IL-4 has been observed to disrupt the functional balance of neurotransmitter systems such as dopamine and norepinephrine; (3) It induces sleep deprivation through intense itching, indirectly worsening cognitive performance; (4) It amplifies systemic inflammation through GBSA microbiome dysbiosis. Concurrently, ADHD-related behavioral abnormalities and psychological stress can also upregulate skin IL-4 expression, forming a bidirectional “skin-brain” interaction mechanism. However, the above hypotheses are primarily based on preclinical studies and indirect observations. whether they can be directly extrapolated to pediatric patients with co-occurring AD and ADHD remains to be validated by large-scale, prospective clinical studies (Figure 2).

Figure 2: A comprehensive diagram illustrating the complex, bidirectional cross-talk along the gut-brain-skin axis, linking intestinal dysbiosis to systemic inflammation, skin manifestation, and neurobehavioral outcomes. (Bottom) At the gut level, microbiota dysbiosis leads to altered metabolism, characterized by reduced production of SCFAs which are subsequently transported into systemic circulation. Intersections with gut sensory neurons are also depicted. (Middle) Systemic circulation acts as a conduit for the transport of SCFAs, IL-4, neutralizing antibodies, and other mediators. These can cross the blood-brain barrier at the vascular-brain interface. (Left) In the skin, features characteristic of AD are shown, including epidermal hyperkeratosis and dermal inflammation involving the cells and mast cells, which are linked to systemic inflammation. Sensory neurons in the skin are involved in itch signaling. (Top) In the CNS, specifically in brain regions like the prefrontal cortex and striatum, circulating IL-4 and potentially other factors (indicated by their receptors) can activate microglia. This activation leads to defective synaptic pruning, a neuronal mechanism potentially underlying comorbid anxiety-like behaviors. The large light blue arrows emphasize the communication within the Gut-Brain Axis. The diagram integrates different spatial and physiological systems with cellular and molecular players.

## 6 Strategies and progress in targeting the IL-4 signaling pathway to intervene in AD comorbidity with ADHD

Current standard treatments for pediatric ADHD only address neurotransmitter imbalances symptomatically and fail to improve the skin inflammation and immune dysregulation associated with comorbid AD. Biologics targeting IL-4/IL-4R*α* have become a core treatment strategy for moderate-to-severe AD. Based on the SBA theoretical framework, systemic blockade of the IL-4 signaling pathway may exert a potential positive effect on neurobehavioral regulation while repairing the peripheral skin barrier, However, its clinical value still requires validation through prospective clinical trials. With the exception of dupilumab—supported by a single retrospective study—direct clinical evidence linking other IL-4/IL-4R*α*-targeted biologics to ADHD is lacking. The following discussion is thus based on theoretical extrapolation from target mechanisms, with representative drugs and their potential associations with ADHD comorbidity summarized in Table 1.

**Table 1:** Exemplary drugs.

Drug Name (Development Code)	Target and Molecular Type	Highest Development Stage for AD	Key Clinical Characteristics	Potential Associations with ADHD Comorbidity	Level of Evidence
I. Single-Target IL-4R*α* Monoclonal Antibody					
1. Approved Drugs					
Dupilumab	IL-4/IL-13, fully human monoclonal antibody	Approved (globally)	First-line standard treatment for moderate-to-severe AD; has the most robust evidence-based data in pediatric populations	Retrospective studies suggest it may reduce the need for stimulant medications in ADHD; the specific mechanism remains to be determined	Retrospective clinical studies
Stapokibart (CM310)	IL-4Rα, humanized monoclonal antibody	Approved (China)	Rapid onset of action, high epidermal repair rate, and good safety profile in children aged 6–11	Good tolerability in the pediatric population, providing a clinical foundation for future studies on comorbidities	Clinical data in pediatric AD + indirect extrapolation
2. Drugs in Phase III Clinical Trials					
Rademikibart (Rademikibart/CBP-201)	IL-4Rα, fully human monoclonal antibody	Phase III	Rapid onset of action, flexible dosing, and superior response in patients with high eosinophil counts	Eosinophil regulation may be indirectly associated with neurodevelopmental abnormalities	Clinical data in pediatric AD + indirect inferences
Talquetamab (GR-1802)	IL-4Rα, fully human monoclonal antibody	Phase III	Outstanding efficacy in patients with concomitant sinusitis and nasal polyps	Improves nocturnal airway ventilation and mitigates the negative impact of sleep deprivation on cognitive function	Clinical data in pediatric AD + indirect extrapolation
Comekibart (Comekibart/MG-K10)	IL-4Rα, humanized monoclonal antibody	Phase III	Long-acting formulation; dosing interval extended to once every 4 weeks	Reduces injection-related anxiety and mitigates the exacerbation of skin inflammation caused by psychological stress	Clinical data in pediatric AD + indirect extrapolation
QX005N	IL-4Rα, monoclonal antibody	Phase III	Approved for the treatment of nodular prurigo	More targeted for the subgroup of severely affected children with chronic, intense scratching	Clinical data on pediatric atopic dermatitis + indirect extrapolation
Other Phase III single-target monoclonal antibodies (SSGJ-611, TQH2722, SHR-1819, mandocizumab, etc.)	IL-4Rα, humanized monoclonal antibody	Phase III	Optimized dosing schedule, partially addressing respiratory comorbidities	Improves sleep through anti-inflammatory and anti-pruritic effects, indirectly alleviating cognition-related symptoms	Clinical data from pediatric AD + indirect extrapolation
II. Multi-target synergistic antibodies					
1. Drugs in Phase II Clinical Trials					
IL-4Rα/IL-31 bispecific antibody (NM26-2198, BBT001)	IL-4Rα/IL-31, bispecific antibody	Phase II	Simultaneously blocks inflammatory pathways and neurogenic itching	Effectively breaks the “itching–sleep disruption–attention deficit” cascade	Theoretical Mechanism Hypothesis
IL-4Rα/IL-33 bispecific antibody (ZW1528, AK139)	IL-4Rα/IL-33, bispecific antibody	Phase II	Upstream Blockade of the Inflammatory Cascade	Long-term unknown risks to pediatric neurodevelopment exist; clinical translation requires extreme caution	Theoretical Mechanism Hypothesis (including safety warnings)
IL-4Rα/TSLP bispecific antibody (IBI3002, BET206-01)	IL-4Rα/TSLP, bispecific antibody	Phase II	Synergistic benefits in patients with airway hyperresponsiveness, such as asthma	Controls systemic atopic inflammation and reduces exposure to central inflammation	Theoretical Mechanism Hypothesis
IL-4Rα/IL-5 bispecific antibody (RC1416)	IL-4Rα/IL-5, bispecific antibody	Phase II	Synergistic regulation of eosinophil function	Intervening in the meningeal immune microenvironment to indirectly influence neurodevelopmental processes	Theoretical Hypothesis on Mechanism
Trispecific antibodies (PF-07275315, PF-07264660)	IL-4/IL-13 + Upstream Alarms, Trispecific Antibodies	Phase II	Comprehensive blockade of the inflammatory cascade	Potent anti-inflammatory effects accompanied by neurodevelopmental risks associated with the corresponding targets	Theoretical Mechanism Hypothesis
2. Drugs in the preclinical research stage					
Preclinical multi-target pipeline (GB12-09, PM1268, SIM0708, etc.)	IL-4Rα multi-target combination	Preclinical	Validation limited to the mechanism level	Possesses only basic exploratory value; no basis for clinical translation	Mechanism-based theoretical hypotheses (preclinical)
III. Biosimilars					
Dupilumab biosimilars (QL2108, GS101, BAT2406, etc.)	IL-4/IL-13, fully human monoclonal antibodies	Phase III/Preclinical	Consistent efficacy and safety with the originator drug	Benefits can be extrapolated to the originator drug; no distinct comorbidity mechanisms	Analogy (biosimilars)

### 6.1 Clinical evidence for single-pathway blockade

IL-4R*α* monoclonal antibodies, represented by Dupilumab,are the standard first-line treatment for moderate-to-severe AD (59). A real-world retrospective study showed that AD children treated with this drug exhibited a significant reduction in the need for ADHD stimulant medications over a 1–5 year period (60). However, it should be emphasized that these observations cannot be directly attributed to the neuroimmune effects of IL-4 blockade; indirect mechanisms such as itch relief, improved sleep, and reduced psychological stress may also be involved, and the relative contributions of these factors require verification through prospective controlled studies. Furthermore, stapokibart (CM310) has demonstrated a more rapid onset of action and a higher epidermal repair rates (61–63), and has has demonstrated good safety in children aged 6–11 (64). providing a feasible foundation for future studies in children with comorbidities. The remaining IL-4R*α* monoclonal antibodies that are either already on the market or in Phase III clinical trials (such as ledecibart and telecibart) have all demonstrated efficacy and safety in pediatric AD populations, with improvements observed in eosinophil regulation (65), and improvement of sinusitis (66), respectively. Their potential benefits for AD children with comorbid ADHD are based on indirect logical inferences regarding “anti-inflammatory effects, itch relief, and improved sleep”, but are not yet supported by direct clinical evidence.

### 6.2 Potential benefits of pharmacokinetic optimization and dosage convenience

Long-acting formulations, such as Comekibart (MG-K10) (67), have reduced the dosing frequency to once every 4 weeks by effectively prolonging the half-life. Theoretically, this could reduce the anticipatory anxiety caused by frequent injections, which holds potential value for pediatric patients with comorbid ADHD and may prevent psychological stress from exacerbating skin inflammation through a feedback loop. Additionally, several IL-4R*α* monoclonal antibodies in clinical development, such as SSGJ-611 (68), TQH2722 (69), and QX005N (69), not only optimize dosing intervals but also demonstrate potential synergistic treatment in patients with concomitant respiratory conditions. Among these, QX005N has been approved for the treatment of Prurigo Nodularis and is particularly targeted toward a subgroup of severely affected pediatric patients who experience prolonged, intense scratching (69). Meanwhile, the development of drugs such as SHR-1819 (70) and Manfidokimab (AK120) (71) has further expanded treatment options for pediatric patients. However, their value in comorbid conditions remains theoretical.

### 6.3 Theoretical mechanisms and potential risks of multi-target synergistic strategies

Given the heterogeneity of ADHD pathogenesis, multi-target synergistic blockade strategies provide a heoretical framework for comorbid intervention. Bispecific antibodies that synergistically block IL-4R*α* and IL-31 [e.g., NM26-2198 (72)] are expected to disrupt the “itching-sleep disruption-attention deficit” cascade by effectively controlling neurogenic itching. Respectively, Bispecific antibodies targeting IL-4R*α*/TSLP and IL-4R*α*/IL-5, offer more targeted intervention strategies for subgroups with comorbid asthma (73) and meningeal immune abnormalities (74). However, clinical evidence for these multi-target approaches remains in the early stages, and their efficacy and safety in patients with comorbidities still require systematic validation. Furthermore, bispecific or trispecific antibodies targeting IL-33 (such as ZW1528) warrant close attention. Since endogenous IL-33 plays a role in maintaining CNS homeostasis and astrocyte-mediated synaptic pruning, systemic blockade during the critical window of neurodevelopment in children carries unpredictable risks. Currently, there are no pediatric safety data or biomarkers to monitor IL-33-related CNS effects; therefore, clinical translation should proceed with extreme caution (75). At the same time, multi-target drugs in the preclinical stage are still in the mechanism-of-action exploration phase, and their value for clinical translation must be evaluated in practice.

### 6.4 ADAs and risks associated with pediatric Use

The immunogenicity of therapeutic monoclonal antibodies may affect pharmacokinetics and efficacy, but this risk is generally manageable with IL-4R*α* monoclonal antibodies. Taking dupilumab as an example, it was well tolerated in the short term among children with AD aged 6 months to 11 years, with common adverse reactions including injection site reactions, conjunctivitis, and herpesvirus reactivation (76). The anti-drug antibodies (ADAs) generated are mostly low-titer and transient, with no significant clinical impact (77). Children's immune systems are not yet fully mature. Currently, there is a lack of large-scale, long-term monitoring data regarding the long-term patterns of ADA development, as well as the long-term effects of prolonged the pathway blockade on parasitic defense, antiviral immunity, and neurodevelopment (78, 79). For children with comorbid AD and ADHD, clinical decision-making must thoroughly weigh the benefits for skin symptoms against potential long-term risks, and long-term follow-up and safety monitoring must be conducted in accordance with established protocols.

## 7 Limitations and outlook

For a long time, the high comorbidity of AD and ADHD in children has often been viewed as the coincidental coexistence of two independent diseases, or simply attributed to the indirect consequences of sleep disturbances caused by itching. Based on the SBA theory, this review proposes the hypothesis that IL-4 may serve as a candidate messenger linking skin inflammation in AD to neurodevelopmental abnormalities in ADHD. Although this hypothesis has a certain degree of biological plausibility, translating basic findings into clinical applications still faces multiple challenges.First, the core causal chain has not yet been established. Current evidence supporting a role for IL-4 in AD-ADHD comorbidity consists of indirect associations and is subject to numerous confounding factors, making it impossible to directly extrapolate these findings to pediatric patients. Second, the SBA theory currently lacks direct *in vivo* evidence of the transmission of cutaneous inflammatory signals to the CNS, as well as quantitative assessment of relevant biomarkers. Third, the safety risks associated with multi-target treatment strategies in children require close vigilance.

Therefore, future efforts should focus on the following key directions: Firstly, prioritize prospective randomized controlled trials with standardized neurocognitive assessments and neuroimaging as co-primary endpoints to clarify the true neurological benefits of interventions. Secondly, utilize multi-omics technologies to identify peripheral biomarkers (such as exosomal microRNAs and dynamic changes in serum IL-4/IL-13 levels) that can predict central effects, thereby providing a basis for precise patient stratification. Thirdly, establish long-term longitudinal cohorts starting in infancy to explore the regulatory role of early inflammation control on neurodevelopmental trajectories and identify the optimal intervention window. In summary, if these approaches are validated, they will facilitate a holistic examination of cross-system comorbidity from a holistic SBA perspective, and provide new directions for the coordinated diagnosis and treatment of AD and ADHD.

## Data Availability

The original contributions presented in the study are included in the article/Supplementary Material, further inquiries can be directed to the corresponding author.
